# Leptospiral flagellar sheath protein FcpA interacts with FlaA2 and FlaB1 in *Leptospira biflexa*

**DOI:** 10.1371/journal.pone.0194923

**Published:** 2018-04-10

**Authors:** Yuya Sasaki, Akihiro Kawamoto, Hajime Tahara, Kie Kasuga, Ryoichi Sato, Makoto Ohnishi, Shuichi Nakamura, Nobuo Koizumi

**Affiliations:** 1 Graduate School of Bio-Applications & Systems Engineering, Tokyo University of Agriculture and Technology, Koganei, Tokyo, Japan; 2 Department of Bacteriology I, National Institute of Infectious Diseases, Shinjuku, Tokyo, Japan; 3 Graduate School of Frontier Biosciences, Osaka University, Suita, Osaka, Japan; 4 Department of Applied Physics, Graduate School of Engineering, Tohoku University, Sendai, Miyagi, Japan; 5 Faculty of Pharmaceutical Sciences, Niigata University of Pharmacy and Applied Life Sciences, Niigata, Niigata, Japan; 6 Division of Medical Sciences, Kanazawa University Graduate School of Medicine, Kanazawa, Ishikawa, Japan; University of Kentucky College of Medicine, UNITED STATES

## Abstract

*Leptospira* spp. are spirochete bacteria that possess periplasmic flagella (PFs) underneath the outer membrane; each flagellum is attached to each end of the protoplasmic cylinder. PFs of *Leptospira* have a coiled shape that bends the end of the cell body. However, the molecular mechanism by which multiple flagellar proteins organize to form the distinctively curled PF of *Leptospira* remains unclear. Here we obtained a slow-motility mutant of *L*. *biflexa* MD4-3 by random insertion mutagenesis using a *Himar1* transposon. In MD4-3, the gene encoding the flagellar sheath protein, flagellar-coiling protein A (FcpA), which was recently identified in *L*. *interrogans*, was inactivated. As with *L*. *interrogans* Δ*fcpA* strains, the *L*. *biflexa* Δ*fcpA* strain lacked a distinct curvature at both ends of the cell body, and its motility was significantly reduced as compared with that of the wild-type strain. PFs isolated from the Δ*fcpA* strain were straight and were thinner than those isolated from the wild-type strain. Western blot analysis revealed that flagellar proteins FlaA1, FlaA2, FlaB1, and FlaB2 were expressed in the Δ*fcpA* strain but the flagellar proteins, except for FlaB2 were not incorporated in its PFs. Immunoprecipitation assay using anti-FcpA antiserum demonstrated that FcpA associates with FlaA2 and FlaB1. The association between FcpA and FlaA2 was also verified using pull-down assay. The regions of FlaA2 and FlaB1 interacting with FcpA were determined using a bacterial two-hybrid assay. These results suggest that FcpA together with FlaA2, produces coiling of PF of the *Leptospira*, and the interaction between the sheath and core filament may be mediated by FcpA and FlaB1.

## Introduction

The genus *Leptospira* is a member of Spirochaetes, having a thin (~140 nm in width), long (~10 μm in length), and coiled cell body and periplasmic flagella (PFs) located beneath the outer membrane. *Leptospira* contains pathogenic, intermediate, and saprophytic species; the pathogenic and intermediate species are etiological agents of worldwide zoonotic leptospirosis [1]. The motility of *Leptospira* spp. facilitated by PFs is an essential virulence factor as some motility-deficient mutants are considerably attenuated [1–3]. Bacteria having external flagella, such as *Escherichia coli* and *Salmonella* spp., swim by rotating helical flagellar filaments similarly to the movements of a screw propeller; each flagellar filament is linked to a rotary nanomachine called the flagellar motor [4, 5]. Although the PF filament of *Leptospira* is also connected to the flagellar motor embedded in the peptidoglycan layer and the cytoplasmic membrane [6], PF rotation within the periplasmic space rotates the protoplasmic cylinder (PC) and periodically transforms the cell envelope, thereby propelling the cell [7–9].

Most of the bacteria belonging to the phylum Spirochaetes have multiple PFs at each cell end, and PFs extending from both cell ends overlap at the center of the cell body. Long PFs in *Borrelia burgdorferi* also function as a cytoskeleton, determining the flat-wave configuration of the cell body [10]. *Treponema phagedenis* also has multiple PFs at each cell end, but they are short (~2.5 μm) as compared to the cell length (~15 μm), and therefore, only the ends are bent by PFs [11]. *Leptospira* spp. possess one PF at each end of the cell body (two PFs per cell), and as observed with *T*. *phagedenis*, leptospiral PFs are not long enough to contact each other. PFs of *Leptospira* bend only the ends of the cell body to form either a spiral or a hook shape [12, 13]; further, the cell ends frequently interchange the shapes (i.e., spiral to hook and vice versa), which is likely owing to reversal of the PF rotation [14]. Conversely, the PC seems to be rigid, and its helical parameters are almost constant during swimming (e.g., ~0.6 μm in the helix pitch) [9, 15]. The morphology of the PC of *Leptospira* could be determined by a homolog of actin MreB [16].

Thus, characteristics of PF are species-specific among spirochetes; however, most commonly, a PF is comprised of a core filament and a sheath [2, 13, 17–20]. Genetic, biochemical, and structural insights indicate that the core and the sheath consist of FlaB and FlaA proteins, respectively [13]. Based on the nucleotide and amino acid sequences, FlaB has been found to be homologous to the flagellin of other bacteria and is probably secreted through the type III secretion system. Conversely, FlaA is a protein specific to spirochetes, and its N-terminal signal sequence is conserved; the presence of the signal sequence implies that the protein is secreted by the SecA system [21]. Flagellar filaments of *E*. *coli* and *Salmonella* are assembled by polymerization of a single type of flagellin [4, 5]. In spirochetes, the PFs of *B*. *burgdorferi* contains a single FlaB and FlaA [21, 22], but that of other species consists of multiple FlaB and FlaA proteins [21, 23]. For example, the PFs of *Brachyspira hyodysenteriae* consist of three FlaB proteins (FlaB1-3) and a FlaA protein; single deletion of FlaB1, FlaB2, or FlaB3 somewhat alters the morphology of *Brachyspira hyodysenteriae* PFs, whereas deletion of FlaA more strongly impacts PF helix parameters and diameter [19].

*L*. *biflexa*, a saprophytic species, has four *flaB* (*flaB*1-4) genes and two *flaA* (*flaA*1 and *flaA*2) genes in its genome [24]. Picardeau et al. reported that inactivation of the *flaB* gene by insertion of a kanamycin resistance marker completely inhibits the assembly of PFs [25]. In addition, Lambert et al. showed that FlaA proteins have important functions in *L*. *interrogans*, and FlaA-deficient *Leptospira* mutants lack curvature at the ends of the cell body and exhibit reduced motility and virulence [2]. However, unlike other spirochetes, the PF sheath is synthesized even in the absence of FlaA proteins [2], suggesting that the leptospiral FlaA protein’s function in the construction of PFs is partially different from its function in other spirochetes. More recently, Wunder et al. found that the inactivation of a gene encoding a novel 36-kDa protein altered the morphology of PFs of *Leptospira* [3]. Because PFs isolated from the mutant exhibited a straight but not coiled shape, the 36-kDa protein was named flagellar-coiling protein A (FcpA) [3]. Furthermore, electron microscopy showed that PFs lacking FcpA were thinner than wild-type (WT) PFs, indicating that FcpA possibly associates with the sheath [3]. Thus, previous studies have elucidated the structure of the PF in *Leptospira* to a certain extent; however, the molecular mechanism by which multiple flagellar proteins orderly synthesize the distinctively curled PF of *Leptospira* remains unclear.

In the present study, we obtained a slow-motility mutant of *L*. *biflexa* MD4-3 by random insertion mutagenesis using a *Himar1* transposon [26] and determined that the transposon was inserted into an open reading frame of *fcpA*. We characterized the phenotypes of the *L*. *biflexa* Δ*fcpA* mutant, analyzed the PFs of the WT and Δ*fcpA* strains, and examined how the inactivation of *fcpA* affects the expression of flagellar proteins and PF composition in *L*. *biflexa*. Furthermore, the interaction between FcpA and other flagellar proteins was assessed using immunoprecipitation, pull-down and *E*. *coli* two-hybrid assays.

## Materials and methods

### Bacterial strains and culture conditions

*L*. *biflexa* serovar Patoc strain Patoc I was cultured in liquid Ellinghausen–McCullough–Johnson–Harris (EMJH) medium at 30°C [27]. Kanamycin and/or spectinomycin were added at a final concentration of 25 μg/ml, as needed. *E*. *coli* JM109 and Π1, strains for DNA cloning, and β2163, the donor strain for conjugation, were grown in LB medium supplemented with 0.3 mM thymidine (Π1) or 0.3 mM diaminopimelate (β2163) [28]. *E*. *coli* XL1-Blue MRF′, the host strain for the BacterioMatch II two-hybrid system, was grown according to the manufacturer’s (Stratagene) instructions.

### Screening of motility-deficient *L*. *biflexa* mutants by random transposon mutagenesis

Random insertion mutagenesis of *L*. *biflexa* using *Himar1* was conducted by conjugation with *E*. *coli* β2163 harboring pCjTKS2, as described previously [26]. After conjugation, cells were spread on a plate of EMJH soft agar (0.4% agar) containing kanamycin, and about 3000 transconjugants were screened for the selection of smaller colonies. The transposon insertion site was identified using the semi-random PCR technique [26].

### Construction of a complemented strain

For the genomic complementation of the kanamycin-resistant Δ*fcpA* strain, the spectinomycin resistance cassette was amplified from pCjSpLe94 [29], and the amplified product was cloned into the transposable element of the PCR-generated pCjTKS2 by In-Fusion cloning (Clontech). The resulting plasmid pS2SPR was digested with *Asc*I, in which the *fcpA* gene with its 5′- and 3′-untranslated regions amplified from the *L*. *biflexa* strain Patoc I was cloned by In-Fusion cloning. Random insertion mutagenesis by conjugation was performed in the *L*. *biflexa fcpA*- strain, as described above. The transposon insertion site was identified using the semi-random PCR technique with TnS1 and TnSN1 primers instead of TnK1/TnK2 and TnKN1/TnKN2, respectively [26]. The transposon insertion site of the complemented strains was within the coding region of a gene in all obtained strains, which was at 131 bp in the gene LEPBI_I2746 in the E6 strain. Primer sequences used in this study are listed in S1 Table.

### Measurement of *Leptospira* motility

Cells grown in EMJH medium were diluted into motility medium (20 mM potassium phosphate buffer, pH 7.4) and observed under a dark-field microscope (BX53, Olympus, Tokyo, Japan). Microscopic images were acquired using a CMOS video camera (IDP-Express R2000, Photron, Tokyo, Japan) at a frame rate of 60 Hz. Swimming cells were tracked with the image analysis software ImageJ (National Institutes of Health), and the swimming speeds were determined with VBA macros originally developed using Microsoft Excel [30]. Swimming speeds of the strains were statistically compared using Student’s *t*-test.

### Electron microscopy and image processing

Purified PFs were applied onto continuous carbon-coated EM grids and negatively stained with 2% (w/v) uranyl acetate solution. Negatively stained EM images were observed using a JEM-1011 transmission electron microscope (JEOL, Tokyo, Japan) operating at 100 kV using a TVIPS TemCam-F415MP CCD camera (TVIPS, Gauting, Germany). The magnification was calibrated by measuring the layer line spacing of 23.0 Å in the Fourier transform of images of the tobacco mosaic virus. The image pixel size at this magnification was 1.95 Å/pixel.

**Fig 1:**
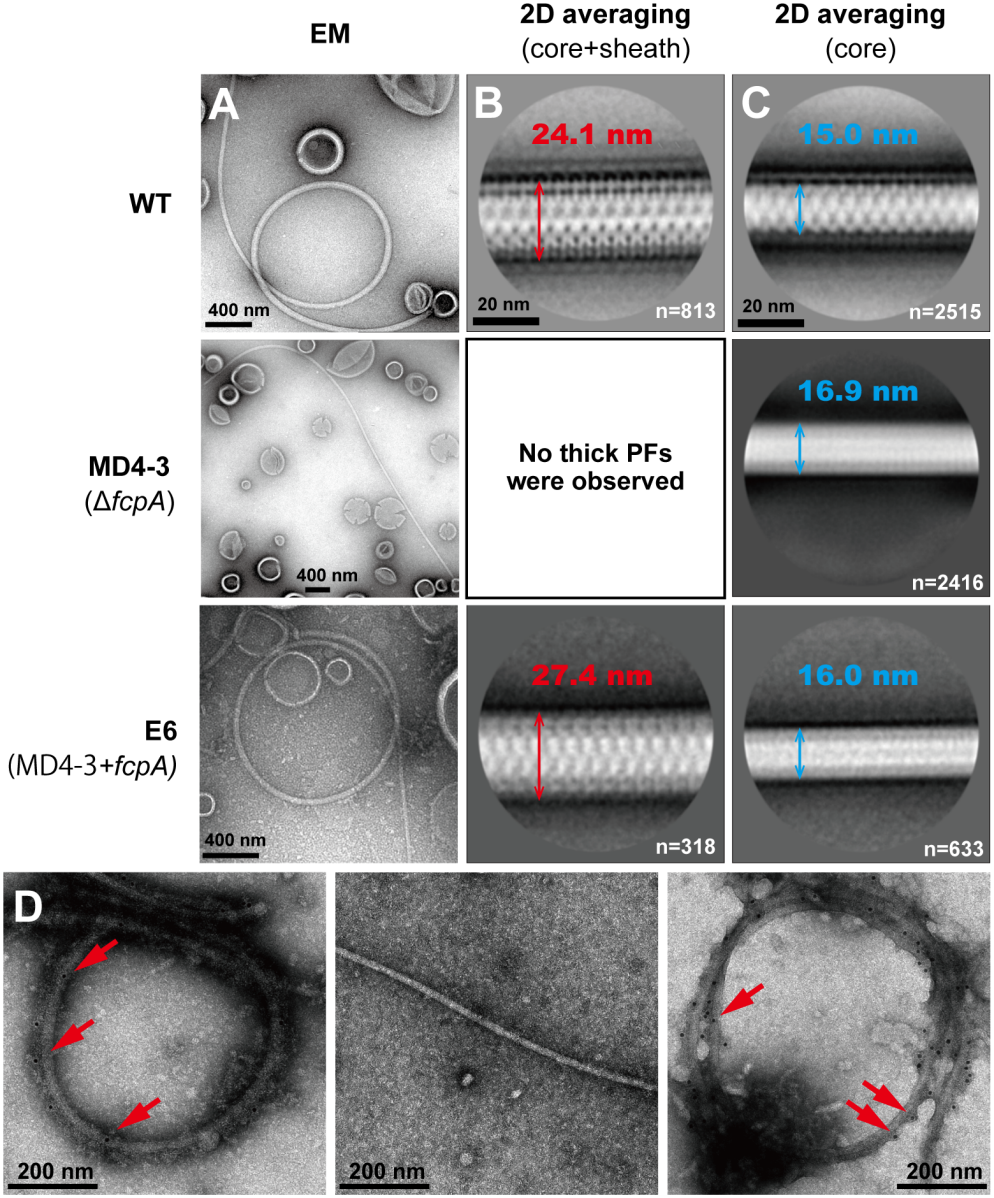
Morphological and immunological characterizations of purified PFs from the wild-type (WT), slow-motility mutant MD4-3 (Δ*fcpA*), and complemented (E6) strains of *L*. *biflexa*. A. Transmission electron microscopic images of negatively stained, purified PFs. B. The diameters of averaged thick PF (core + sheath) images. n: number of particles used for averaging. C. The diameters of averaged thin PF (core) images. n: number of particles used for averaging. D. Immunoelectron microscopic images of purified PFs labeled with anti-FcpA antiserum. Left: WT, middle: MD4-3, right: E6. Arrows indicate 10 nm gold nanoparticles conjugated to secondary antibody.

The total number of extracted particles and micrographs used in the experiments is described in Fig 1B and 1C. The following image analysis was performed with Relion [31]. Individual images of PFs of the WT, motility-deficient mutant MD4-3 (Δ*fcpA*), and complemented (E6) strains were manually selected, and the segments at 400 × 400 pixels were extracted from several dozens of images with a step shift of 40 pixels along the helical axis. Each segment was subjected to reference-free two-dimensional (2D) classification, and only “good” class-averaged images were selected for calculating the helical pitch and diameter.

### Immunoelectron microscopy

Sample solutions suspended in fresh ultrapure water at 10-fold dilution were applied onto continuous carbon-coated EM grids. After excess solution was removed by filter paper, the grids were treated with a primary antibody against FcpA at a 10-fold dilution in 0.1% BSA for 20 min. The grids were washed five times with ultrapure water and then treated with a 50-fold diluted gold-labeled secondary antibody (goat antibody labeled with 10 nm colloidal gold; Sigma) in 0.1% BSA for 20 min. Next, the grids were washed five times with ultrapure water and were then negatively stained with 2% (w/v) uranyl acetate solution. Grids were examined with the JEM-1011 transmission electron microscope operating at 100 kV using the TVIPS TemCam-F415MP CCD camera.

### Purification of PFs, SDS-PAGE and immunoblotting experiments

PFs were purified by the method described by Wunder et al. [3]. Protein samples (1.5 μg of purified PFs, 1.5 × 10^8^ leptospiral cells suspended in SDS-PAGE sample buffer, the immunoprecipitates or bait-prey complex from pull-down assays described below) were subjected to SDS-PAGE and Western blotting. The blot was incubated with antisera raised against peptide fragments of FcpA (NH2-QPANAQESQAAKDQ-COOH and NH2-PENDDAELTEDQKK-COOH), FlaA2 (NH2-SEAKYPGSEIKDNW-COOH), and FlaB1, FlaB2, FlaB3, and FlaB4 (S1A Fig) or anti-FlaA1 serum [32]. The dilutions of antisera were 10000 fold for anti-FcpA and FlaB2 antisera, 3000 fold for FlaB1, FlaB3 and FlaB4 antisera, and 1000-fold dilution for anti-FlaA1 and FlaA2 antisera.

### Immunoprecipitation

Immunoprecipitation was performed as described by Motaleb et al. [33]. Approximately 5×10^9^ *L*. *biflexa* cells were washed twice with phosphate-buffered saline (PBS) containing 5 mM MgCl_2_ and lysed in 20 ml of TSEA buffer (50 mM Tris-HCl, 150 mM NaCl, 5 mM EDTA, and 0.05% sodium azide, pH 7.5) containing 1% Nonidet P-40 and protease inhibitors (cOmplete^™^ protease inhibitor cocktail, Roche) at 37°C for 1 h. The lysate was centrifuged at 4,000×*g* for 30 min at 25°C, and the pellet was resuspended in 1.5 ml of PBS and sonicated for 30 s 40 times in ice/water. The lysate was centrifuged at 14,800×*g* for 30 min at 25°C, and the supernatant was incubated with 1 μl of anti-FcpA antiserum or its pre-immune serum for 1 h at 25°C in the presence of 1% bovine serum albumin. After 1 h, 100 μl of 10% Protein G agarose (Thermo Fisher Scientific) was added to the sample, which was further incubated for 1 h at 25°C. The immunoprecipitates were washed three times with 1 ml of TSEA buffer containing 0.05% Tween-20. The final pellets were suspended in 20 μl of SDS-PAGE sample buffer and then boiled. Finally, 10 μl of the supernatant was used for electrophoresis and Western blotting.

### Pull-down assays

FcpA (aa 22–300) was produced as a glutathione *S*-transferase (GST) fusion proteins. The amplified *fcpA* gene was cloned into *Bam*HI- and *Sal*I-digested pGEX-6P-1 (GE Healthcare), and then transformed into *E*. *coli* JM109. Transformed *E*. *coli* cells were grown at 37°C in LB medium containing ampicillin (50 μg/ml) overnight, and the culture was diluted into fresh medium at 1: 10 and incubated for 1 hr at 37°C followed by 5 hr-incubation at 25°C in the presence of IPTG (final conc. 0.1 mM). The cells were disrupted as described previously [34]. About 5 μg of recombinant GST/FcpA fusion proteins or GST was immobilized on glutathione Sepharose 4B (GE Healthcare), which was incubated with 500 μl of WT cell lysates prepared as described above for 2 hr at 4°C. The beads were washed four times with PBS and the final pellets were suspended in 20 μl of SDS-PAGE sample buffer and then boiled. Finally, 10 μl of the supernatant was used for Western blotting.

### Bacterial two-hybrid assays

Two-hybrid assays in *E*. *coli* were conducted using the BacterioMatch II two-hybrid system (Stratagene). The amplified *fcpA* gene was cloned into *Xho*I- and *Not*I-digested pBT, and the amplified *flaA2* and *flaB1* genes and their truncated derivatives were individually cloned into *Xho*I- and *Not*I-digested pTRG. The full-length genes were also cloned into reverse vectors. Pairs of the bait and prey plasmids were co-transformed into *E*. *coli* XL1-Blue MRF′, and the transformants were selected on an M9 medium plate containing chloramphenicol (25 μg/ml), tetracycline (12.5 μg/ml), and 5 mM 3-amino-1,2,4-triazol (3-AT), followed by further selection on an M9 medium plate containing the above reagents plus streptomycin (12.5 μg/ml).

## Results

### Characterization of the motility-deficient *L*. *biflexa* mutant MD4-3 (Δ*fcpA* mutant) and its PFs

**Fig 2:**
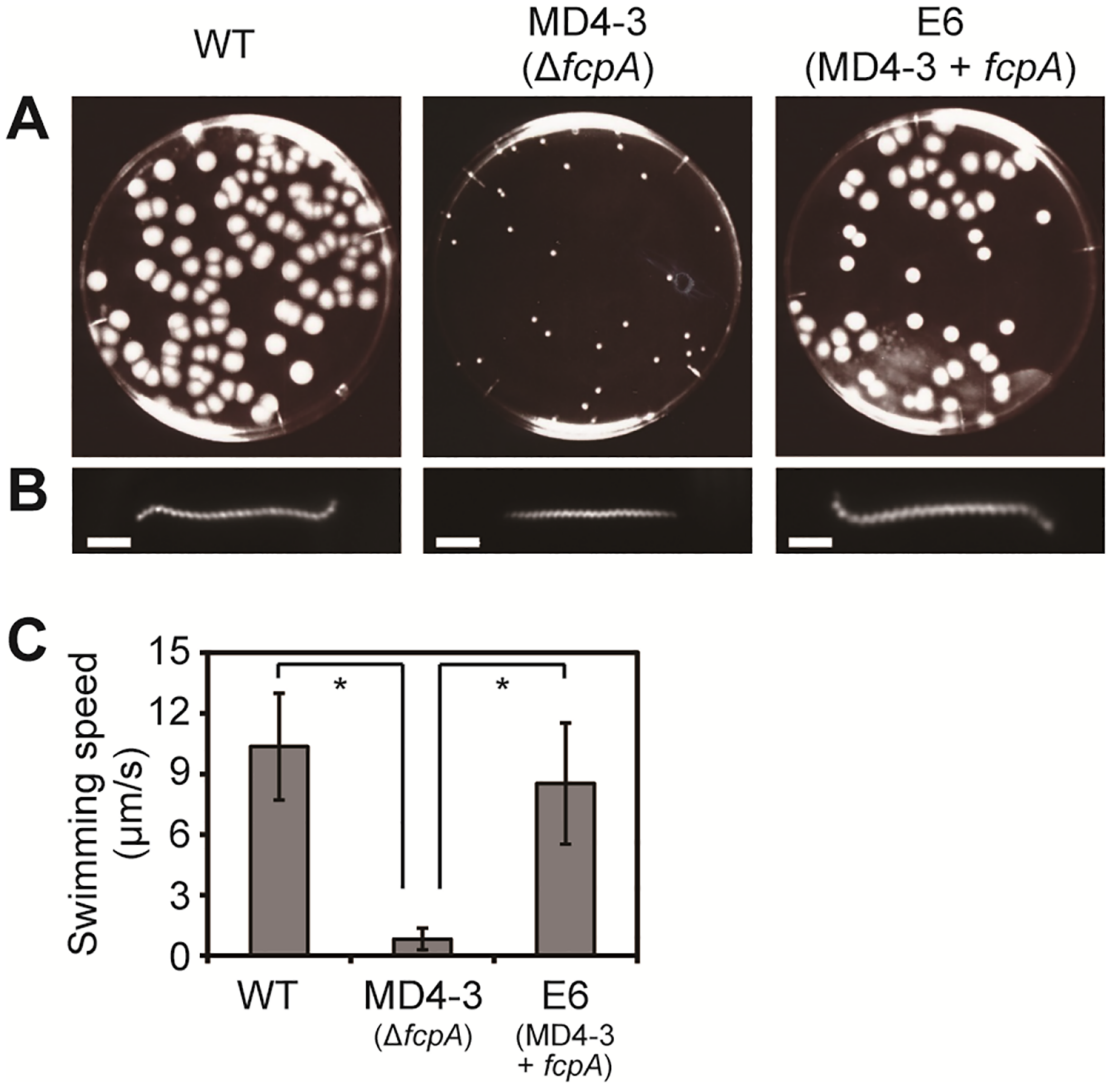
Phenotypes of wild-type (WT), slow-motility mutant MD4-3 (Δ*fcpA*), and complemented (E6) strains of *L*. *biflexa*. A. Colony formation on a 0.4% agar plate. B. Dark-field micrographs of *L*. *biflexa* cells. Bar = 2 μm. C. Swimming speeds of *L*. *biflexa* strains. *p < 0.001.

Using transposon random mutagenesis, we obtained an *L*. *biflexa* clone MD4-3 that formed a smaller colony than the WT strain on 0.4% agar plate (Fig 2A). MD4-3 had a transposon insertion in *fcpA* (718 bp into the 903-bp gene), which has recently been identified as a gene for a flagellar sheath protein in *L*. *interrogans* serovar Copenhageni [3]. Dark-field microscopy showed that both cell ends of WT cells had a curved shape, whereas those of the Δ*fcpA* cells lacked the distinctive curvature (Fig 2B). The WT strain swam at 10.4 ± 2.6 μm/s (mean ± SD; n = 28 cells) (Fig 2C). On the other hand, the movement of the Δ*fcpA* strain was significantly slower than that of the WT strain (p < 0.001), but the strain was still able to swim at 0.8 ± 0.5 μm/s (mean ± SD; n = 14 cells) (Fig 2C). Such effects of the *fcpA* inactivation on the cell morphologies and the motility of *L*. *biflexa* are consistent with those observed in Δ*fcpA* strains of *L*. *interrogans* [3]. Genomic complementation of the Δ*fcpA* strain restored the colony size as well as the cell morphology (Fig 2A and 2B). The swimming speed of the complemented strain was significantly faster than that of the Δ*fcpA* strain (p < 0.001, Fig 2C).

As with *L*. *interrogans* Δ*fcpA* strains [3], transmission electron microscopic images of negatively stained, purified PFs revealed straightened PFs from the Δ*fcpA* strain as opposed to coiled PFs from the WT strain (Fig 1A). Genomic complementation of the Δ*fcpA* strain restored coiled PF (the bottom panel of Fig 1A). As can be seen in Fig 1B and 1C, which depict the averaged electron cryomicrographs of PFs isolated from WT, Δ*fcpA* and the complemented strains, PF diameters from WT and Δ*fcpA* strains were ~25 and 15 nm, respectively. It is noteworthy that the complementation restored the diameter to almost the same value as the WT. These results suggest that PFs from the Δ*fcpA* mutant lack a sheath. Most WT PFs were thicker than ΔfcpA mutant PFs, but some WT PFs were as thin as ΔfcpA mutant PFs (Fig 1B). Therefore, thin WT PFs reveal that PFs could have been in the process of structuring or could have formed by sheath removal, which occurred artificially during their purification. Immunoelectron microscopy revealed that anti-FcpA antibodies bound to purified PFs from the WT strain but not to those from the Δ*fcpA* strain (Fig 1D). Genomic complementation also restored the expression of FcpA on the surface of PFs (the bottom panel of Fig 1D).

### Comparison of the expression of flagellar proteins in WT and Δ*fcpA* strains

*L*. *biflexa* has four *flaB* genes [24], but the levels of their expression in *L*. *biflexa* are unclear. Antisera were raised against a peptide fragment from each FlaB protein (S1A Fig), which specifically reacted with the corresponding recombinant FlaB proteins, although slight cross-reactions of the anti-FlaB1 serum with FlaB2 and the anti-FlaB3 serum with FlaB2 were observed (S1B Fig). Western blot analysis revealed bands with the expected molecular masses when probed with anti-FlaB1 and FlaB2 antisera but not with anti-FlaB3 and FlaB4 antisera, both in leptospiral cells and purified PFs (S2 Fig). These results suggest that only FlaB1 and FlaB2 may be expressed in *L*. *biflexa*.

**Fig 3:**
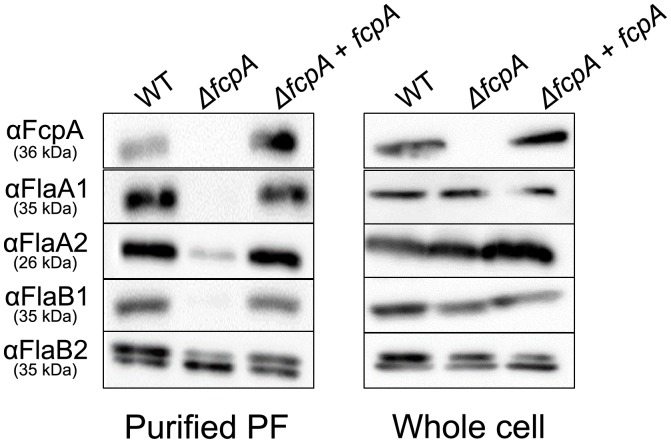
Immunoblot analysis of purified PF (left panel) or whole cell lysates (right panel) from the wild-type (WT), Δ*fcpA* and complemented strains of *L*. *biflexa*. Purified PFs (1.5 μg) or *L*. *biflexa* whole cell lysates (1.5 × 10^8^ cells) were subjected to 10% SDS-PAGE and Western blotting with anti-FcpA, FlaA1, FlaA2, FlaB1, or FlaB2.

Western blot analysis also demonstrated that FlaA1 and FlaB1 were completely absent and FlaA2 was mostly absent in purified PFs from the Δ*fcpA* strain compared to those from the WT strain, although these proteins were expressed in the mutant cells (left and middle lanes in Fig 3). Genomic complementation of the Δ*fcpA* strain restored the detection of FlaA1 and 2 and FlaB1 in the purified PFs (right lane in Fig 3).

### Association of FcpA with FlaA2 and FlaB1

**Fig 4:**
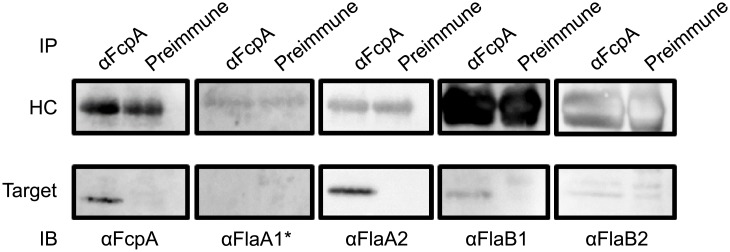
Immunoprecipitation of flagellar proteins with anti-FcpA. Lysates from the wild-type strain were immunoprecipitated with anti-FcpA antiserum and then probed with antisera against FcpA, FlaA1, FlaA2, FlaB1, and FlaB2 by Western blotting. The upper panels indicate the antibody heavy chains (MW: approximately 50 kDa) from the primary antisera or pre-immune sera. *Anti-FlaA1 antiserum was raised in a mouse.

The presence of FlaA1, FlaA2, and FlaB1 in the cells but not in the purified PFs from the Δ*fcpA* strain suggested that these proteins were bound to FcpA and were located in the PF. To investigate the association of FcpA with Fla proteins, FcpA was immunoprecipitated from WT cell lysates with anti-FcpA antiserum. Then, the resulting immunoprecipitates were subjected to immunoblotting with anti-Fla protein antisera. As a result, FlaA2 co-precipitated with FcpA, whereas FlaA1 did not (Fig 4). In addition, overexposure of the blot revealed that FlaB1, but not FlaB2 (although non-specific bands were detected in both antisera and pre-immune fractions), was also co-purified with FcpA, suggesting a weak interaction between FcpA and FlaB1 (Fig 4). The association of FcpA with FlaA2 and FlaB1 was specific, because these Fla proteins did not co-precipitate from Δ*fcpA* cell lysates when anti-FcpA antiserum was used (S3 Fig).

The association between FcpA and FlaA2 was verified using GST pull-down assays while FlaB1 was not recovered from WT cell lysate by GST/FcpA fusion proteins (S4 Fig).

### Regions of FlaA2 and FlaB1 that interact with FcpA

**Fig 5:**
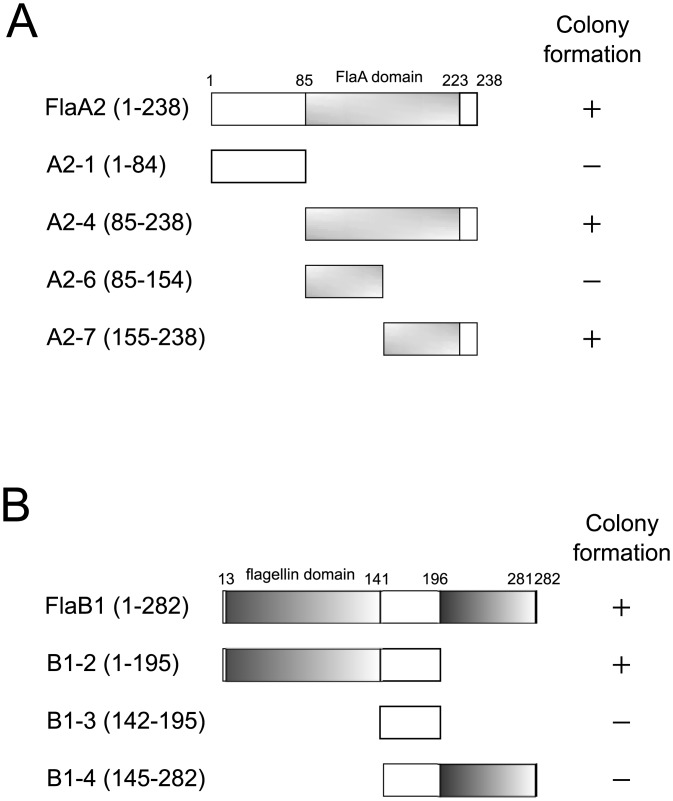
Schematic representation of peptide fragments used in bacterial two-hybrid assays and the growth results of *E*. *coli* expressing FcpA and the FlaA2 derivatives (A) and the FlaB1 derivatives (B) in the selective medium supplemented with 3-AT and streptomycin. No *E*. *coli* transformants were obtained when transformed with the plasmids encoding FlaA2 fragments A2-2 (1–154), A2-3 (1–223), or A2-5 (85–223) and with the plasmid encoding FlaB1 fragment B1-1 (1–144).

To determine the regions of FlaA2 and FlaB1that are involved in the interaction with FcpA, the BacterioMatch II two-hybrid system (Stratagene) was employed in this study. Efficient growth of *E*. *coli* transformants, containing plasmids of *fcpA* as a bait and *flaA2* or *flaB1* as a prey, was observed on the dual selective medium (M9+ medium supplemented with 3-AT and streptomycin) (S5 Fig). Moreover, *E*. *coli* transformants with reverse plasmids of the above genes also displayed efficient growth on the dual selective media (S5 Fig). The truncated derivatives of FlaA2 and FlaB1 were designed based on their secondary structure, and FcpA was found to interact with the aa155–238 region of FlaA2 (Fig 5A) as well as with the aa1–140 region of FlaB1 (Fig 5B).

## Discussion

In this study, we obtained a slow-motility mutant of *L*. *biflexa* by random transposon mutagenesis and found that the gene encoding the flagellar sheath protein FcpA, which has previously been identified in *L*. *interrogans* [3], was inactivated in the mutant. As observed in the Δ*fcpA* mutant of *L*. *interrogans*, our *L*. *biflexa* Δ*fcpA* strain maintained a short-pitch spiral shape of PC but lacked a distinct curvature at both ends of the cell body (Fig 2B). PFs isolated from the Δ*fcpA* strain were straight and were thinner than those from the WT strain (Fig 1). Dual knock-out of *flaA1* and *flaA2* in *L*. *interrogans* did not alter the diameter of the PF [2], whereas the lack of FcpA with partially reduced FlaA2 decreased the thickness of the PFs of *L*. *interrogans* [3]. These results indicate that PF of *Leptospira* contains a sheath, but that neither FlaA1 nor FlaA2 are likely to be major sheath proteins. In both *L*. *interrogans* [3] and *L*. *biflexa* (Fig 1D), immunoelectron microscopy using anti-FcpA antiserum indicated that FcpA is uniformly distributed on the surface of thick PFs (~25 nm in diameter). Although it has not been investigated whether FcpA is associated with PFs in the FlaA1- and FlaA2-deficient *L*. *interrogans* mutant [2], FcpA was one of the major sheath proteins common to *L*. *interrogans* and *L*. *biflexa*. Thus, the effect of *fcpA* inactivation on the morphologies of the cell body and PFs was consistent with *L*. *biflexa* and *L*. *interrogans*, but detailed analyses showed some new insights into PF composition and FcpA function as discussed below.

Although *Leptospira* spp. possess two *flaA* genes and four *flaB* genes in the genome, it has been unknown how these proteins are incorporated into the PF. Malmström et al have demonstrated that all four FlaB proteins are expressed in *L*. *interrogans* cells [23]. In this study, only FlaB1 and FlaB2 were detected in *L*. *biflexa* by Western blot analysis using peptide-specific antisera that exhibited high specificity and reactivity (S1 and S2 Figs). Although we cannot completely negate the possibility of poor avidity of anti-FlaB3 and FlaB4 antisera, our results suggest that the PFs of *L*. *biflexa* consists mainly of FlaB1, FlaB2, FlaA1, and FlaA2. The inactivation of *fcpA* did not affect the expression of these flagellar proteins (Fig 1, right panels) but completely suppressed the association of FlaA1 and FlaB1 with PFs and suppressed mostly that of FlaA2 (Fig 3, left panels). This result demonstrates that *Leptospira* FlaB2 alone can polymerize and form the core filament. In addition, diameters of the thin PF parts from WT were almost the same as those of the PFs from the Δ*fcpA* strain comprising only FlaB2, suggesting that the packing arrangement of FlaB2 in the PFs of Δ*fcpA* was intact. *Brachyspira hyodysenteriae* has three *flaB* genes (*flaB1*, *flaB2*, and *flaB3*); all of which are involved in the formation of completely functional PFs [19, 35, 36]. However, Li et al. showed that the *flaB1 flaB3* double knock-out mutant formed a core filament of PF composed of FlaB2 [36] while *B*. *burgdorferi* has only one FlaB [10]. Thus, multiple spirochete FlaB proteins are unlikely to be necessary for polymerization. However, PFs are not synthesized in the *flaB* (*flaB1* in our study) knock-out *L*. *biflexa* cells [25]. A decrease of FlaA in a *flaB* mutant has been demonstrated in *B*. *burgdorferi*, in which FlaA is associated with FlaB; the absence of FlaB negatively influenced the translational control or protein stability of FlaA [33]. Therefore, the inactivation of *flaB1* in *L*. *biflexa* could influence the translational control or protein stability of FlaB2, similar to that observed in *B*. *burgdorferi*.

Li et al. examined the helicity of PFs isolated from the *flaB1 flaB3* double knock-out mutant of *Brachyspira hyodysenteriae* and found that the helix pitch of PFs composed of FlaB2 and FlaA was almost the same as that of PFs from WT composed of three FlaB proteins and FlaA [36]. However, the inactivation of *flaA* yielded PFs with aberrant helicity even when all sets of FlaB were expressed normally. Furthermore, FlaA was found to solely enable the formation of the sheath in the absence of the core filament, but the sheath structure did not exhibit a helical morphology [36]. These previous results indicate that an interaction between sheath (FlaA) and core (FlaB) proteins is responsible for the regulation of PF helicity in *Brachyspira hyodysenteriae*. The previous study on *L*. *interrogans* [3] and our study on *L*. *biflexa* have shown that FcpA plays a key role in coiling PFs in both *Leptospira* species. Lambert et al. showed that *L*. *interrogans* strains lacking FlaA1 and FlaA2 also synthesize straight PFs although the expression of FcpA was not confirmed in these strains [2]. We found that the inactivation of *fcpA* considerably decreased the amount of FlaA1 and FlaA2 in straight PFs of *L*. *biflexa* (Fig 3). We also found that FcpA interacts with the C-terminal region of FlaA2 and N-terminal region of FlaB1 (Figs 4 and 5). These results raise the possibility that, in *L*. *biflexa*, FcpA bends the PF together with FlaA2 via interaction with FlaB1.

Incorporation of FlaA proteins into the *L*. *biflexa* PF were almost completely inhibited by the inactivation of *fcpA* (Fig 3), but the reduction of FlaA proteins in the PF was moderate in *L*. *interrogans*: compared with the WT strain, 74% of FlaA1 and 42% of FlaA2 were detected in the PFs of the *L*. *interrogans* Δ*fcpA* strain [3]. These facts imply that FcpA is likely to govern incorporation of FlaA proteins into the PF in *L*. *biflexa*, but additional proteins other than FcpA might be involved in the PF construction in *L*. *interrogans*.

In summary, results obtained in this study propose a possible structure of the *L*. *biflexa* PF. The core filament consists mainly of FlaB1 and FlaB2; the basic structure of the sheath is formed by FcpA, associating with FlaA2; the core filament interacts with the sheath via weak binding between FlaB1 and FcpA, determining the coiled shape of the PF. However, there are still some unsolved issues. The weak association between FcpA and FlaB1 observed *in vivo* (in immunoprecipitation assays) was not reproduced by *in vitro* pull-down assays. The FcpA-FlaB1 interaction might be reproducible *in vitro*, but only under specific conditions. Further improvement of experimental conditions in pull-down assays as well as other assays such as surface plasmon resonance could verify their interaction *in vitro*. In this study, double bands of FlaB2 were detected by Western blotting (Fig 3). Although our preliminary experiments ruled-out phosphorylation of FlaB2 (data not shown), which has been observed in the flagellins of *Pseudomonas aeruginosa* [37, 38], the molecular explanation and the biological significance of the doublet FlaB2 remain unknown. The configuration of FlaA1 in PFs remains unclear. Nevertheless, FlaA1 might be associated with FlaA2. More detailed analysis of the molecular interactions between FcpA, possible unidentified proteins, and Fla proteins will contribute to obtaining new insights into the mechanisms of synthesis and shape determination of the spirochete flagellum.

## Supporting information

S1 Fig Locations of peptide fragments (indicated in red) used for the preparation of antiserum for each FlaB protein (A) and the specificity of each anti-FlaB antisera (B). Western blotting was performed with anti-FlaB antisera against recombinant FlaB proteins. Lanes M, molecular weight marker; 1, *E*. *coli* lysate expressing rFlaB1/GST; 2, *E*. *coli* lysate expressing rFlaB2/His; 3, *E*. *coli* lysate expressing rFlaB3/His; 4, *E*. *coli* lysate expressing rFlaB4/GST. Asterisks indicate recombinant FlaB proteins.
(PDF)

S2 Fig Reactivity of anti-FlaB sera against *L*. *biflexa* whole cell lysate and Purified PFs. Western blotting was performed with anti-FlaB antisera (upper panel) and anti-FlaA2 serum (lower panel). The number of the lanes on the upper panel indicates anti-FlaB1, 2, 3, and 4 antisera, respectively.
(PDF)

S3 Fig Immunoprecipitation of flagellar proteins with anti-FcpA. Lysates from the wild-type and the Δ*fcpA* strains were immunoprecipitated with anti-FcpA antiserum and then probed with antisera against FcpA, FlaA2 and FlaB1 by Western blotting. The upper panels indicate the antibody heavy chains (MW: approximately 50 kDa) from the primary antisera.
(PDF)

S4 Fig Pull-down of FlaA2 by GST/FcpA fusion proteins. Lysates from the wild-type strain were subjected to pull-down assay with GST/FcpA fusion proteins or GST and then probed with anti-FlaA2 antiserum by Western blotting (A). The same blot was stained with Ponceau S (B).
(PDF)

S5 Fig Two-hybrid analysis of the interaction between FcpA and FlaA2 (A) and FlaB1 (B). Representative plates showing the growth of *E*. *coli* transformants. (I) bait (b): FlaA2; target (t): FcpA, (II) b: FcpA; t: FlaA2, (III) b: FlaA2; t: empty, (IV) b: FcpA; t: empty, (V) b: FlaB1; t: FcpA, (VI) b: FcpA; t: FlaB1, (VII) b: FlaB1; t: empty, (VI) b: FcpA; t: empty.
(PDF)

S1 Table Primer sequences used in this study. Underlining indicates a restriction enzyme site.
(XLSX)
