# Supplementary material for: Leptospiral flagellar sheath protein FcpA interacts with FlaA2 and FlaB1 in Leptospira biflexa
Source: PLoS One. 2018 Apr 10;13(4):e0194923. doi: 10.1371/journal.pone.0194923 (PMC5892894; doi:10.1371/journal.pone.0194923)
Supplement: S1 Fig — Western blotting was performed with anti-FlaB antisera against recombinant FlaB proteins. Lanes M, molecular weight marker; 1, E. coli lysate expressing rFlaB1/GST; 2, E. coli lysate expressing rFlaB2/His; 3, E. coli lysate expressing rFlaB3/His; 4, E. coli lysate expressing rFlaB4/GST. Asterisks indicate recombinant FlaB proteins. (PDF) [file pone.0194923.s001.pdf]

A

```
FlaB1   1:--MIINHNVSAIFAHRTLKSNDANLSKDIEKLSSGMRINKAGDDASGLAVSEKMRTQIAG 58
FlaB2   1:--MIINHNLAAINSHRVLKFQNEEVSKNMEKLSSGMRINRAGDDASGLAVSEKMRTQVNG 58
FlaB3   1:--MIINHNMSAIQSHRALKFTQWDVDKTMRNLSTGQRINLAGDDASGLAVSEKLRTQIRG 58
FlaB4   1:VTMIINHNISALVAKRALSNTSRDMDKSMEHLATGMRINRPGDDSLGFSVSEKLRSQIRA 60
            ****** .*.  .* *.       * .. *. *.*** .***..*..**** *.*. .

FlaB1  59:LRRAEQNTEDGMSLIQTAEGYLQETHEIVQRVRVLAVQAANGIYSEEDRQQIQVEVSQLV 118
FlaB2  59:LRQAERNTEDGMSLIQTTEGFLQESNDIIQRIRTLAIQSSNGIYTEEDRQMIQVEVSQLI 118
FlaB3  59:LRQAERNTEDGLSFIQTAEGYLDQSAEIIQRIRTLAIQTSNGIYTPEDRQLVQVEVSALV 118
FlaB4  61:LGQAERNTQDGMSFLQVTEGSLDQVNSILQRLRELSVQSANGIYSDEDRKLVQLEVSQLV 120
          *..**.**.**.* .*. ** *     * ** * *. *  ****  ***.  *.***.*.

FlaB1 119:DEIDRIASQAEFNKMKLLTGAFARLNPTASMWFHIGANMHQRERVYIETMNTAALGLRNP 178
FlaB2 119:DEVDRIASQAEFNKMNLLQGDFARGSRATSMWFHIGPNMHQRERVFIATMTARSLNLKGQ 178
FlaB3 119:DEIDRIASQAEFNKMKLFEGDFARKSTKASMWFHMGANARQRERFYIGTMTSKALKMSEG 178
FlaB4 121:EEVERIGTSAEFNKVKPLDGRFSRAN-KNPMTLQVGANGTEKIEVYINTMTSSSLKLKQA 179
          .* .**...*****.... * *.*     .*... *.*  ......* **.   * .

FlaB1 179:TVLTFISLSTAGKANSVIGLCDDALRVISKQRADLGAYYNRMEHAAKGLMNAYENTQASE 238
FlaB2 179:SG-ELLSLSTADKSNDAIGTLDAALTRISKQRANLGAYFNRLEHAAKGLMNAYENTQASE 237
FlaB3 179:AI--KIALSTPGKADEAIAKADFALNKIMKQRADMGAYQNRLESTAKGLMGAYENMQASE 236
FlaB4 180:GN--KLTLSTPNKASDSLQVLDDAISKVNRLRSDLGAYYNRLDLTLKSLSNNYVNMVSSE 237
                 ***  *.   .   * *.  . ..*...*** **..  .*.*...*.* ..**

FlaB1 239:SRIRDTDMAEQMTSFTRYQILTQAATSMLAQANMKSQSVMRLLQ---         282
FlaB2 238:SRIRDADMAEETVAFTKNQILVQSGTAMLAQANVRPQGVLSLLR---         281
FlaB3 237:SRIRDADMAEEMVALTTKQILVQSGTAMLAQASLRPNSVLRLLNNT-         282
FlaB4 238:SQIRDADMATEMVEYSKNQILTKSGVAMLAQANLRPESVVKLLTDRY         284
          *.***.***....  .  *** .....*****. .. .*  **
```

B

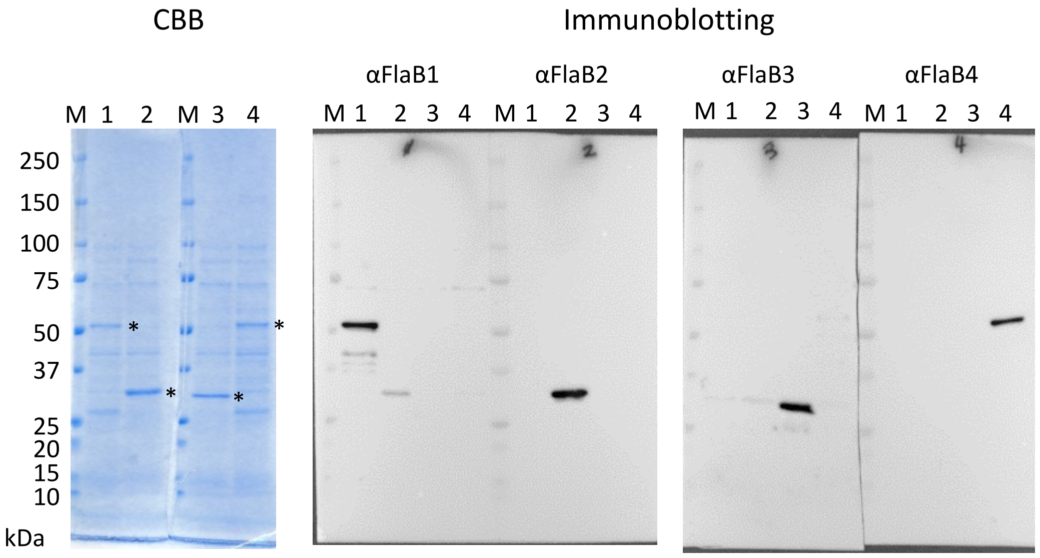

S1 Fig. Locations of peptide fragments (indicated in red) used for the preparation of antiserum for each FlaB protein (A) and the specificity of each anti-FlaB antiserum (B).
