# Supplementary figures and images for: Leptospiral flagellar sheath protein FcpA interacts with FlaA2 and FlaB1 in Leptospira biflexa
Source: PLoS One. 2018 Apr 10;13(4):e0194923. doi: 10.1371/journal.pone.0194923 (PMC5892894; doi:10.1371/journal.pone.0194923)

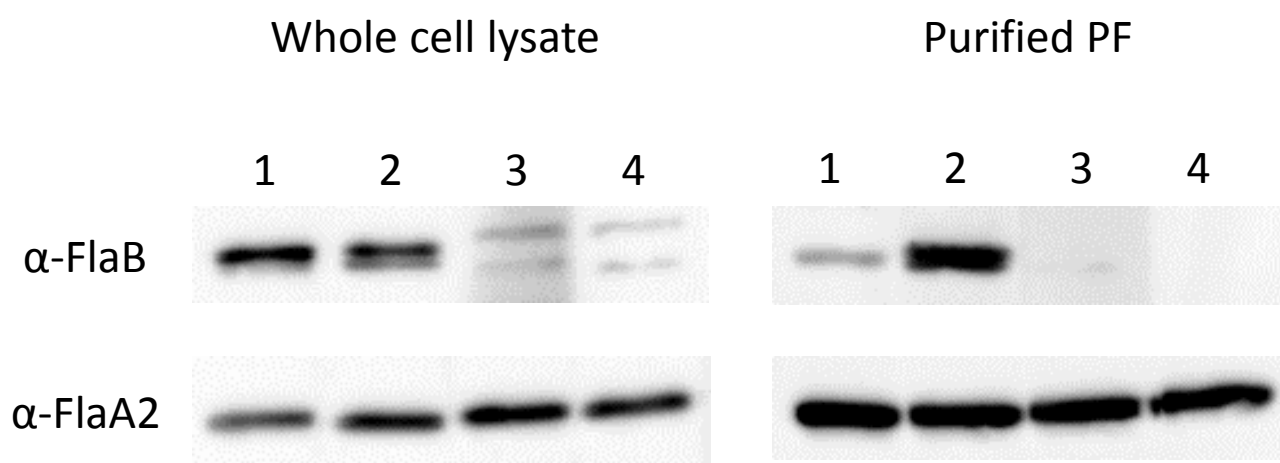

**S2 Fig. Reactivity of anti-FlaB sera against *L. biflexa* whole cell lysate and Purified PFs.**

Supplement: S2 Fig — Western blotting was performed with anti-FlaB antisera (upper panel) and anti-FlaA2 serum (lower panel). The number of the lanes on the upper panel indicates anti-FlaB1, 2, 3, and 4 antisera, respectively. (PDF) [file pone.0194923.s002.pdf]

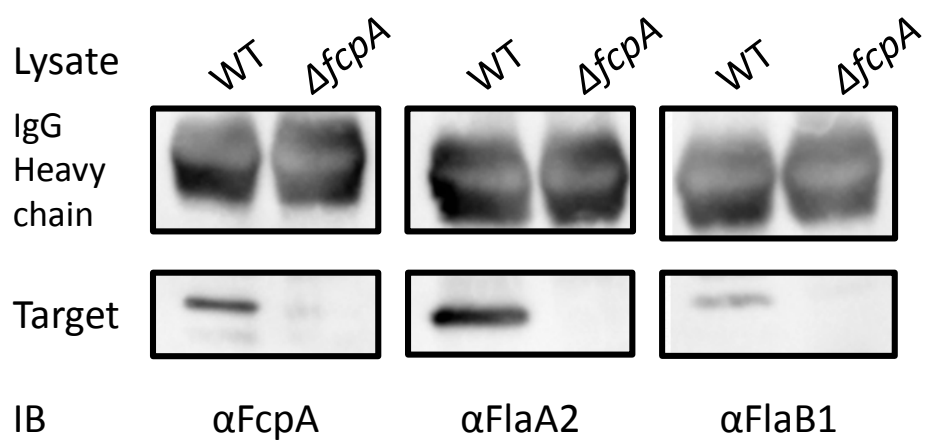

**S3 Fig. Immunoprecipitation of flagellar proteins with anti-FcpA.**

Supplement: S3 Fig — Lysates from the wild-type and the ΔfcpA strains were immunoprecipitated with anti-FcpA antiserum and then probed with antisera against FcpA, FlaA2 and FlaB1 by Western blotting. The upper panels indicate the antibody heavy chains (MW: approximately 50 kDa) from the primary antisera. (PDF) [file pone.0194923.s003.pdf]

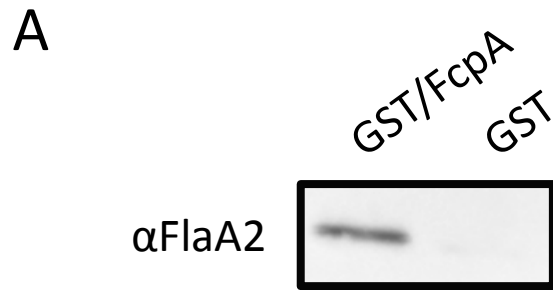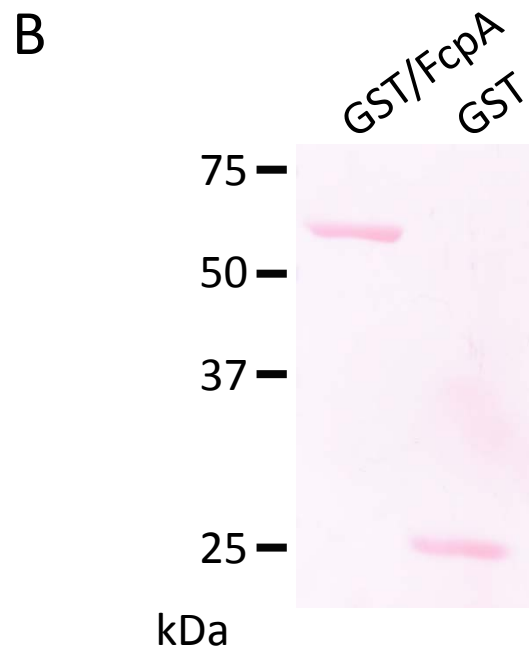

**S4 Fig. Pull-down of FlaA2 by GST/FcpA fusion proteins.**

Supplement: S4 Fig — Lysates from the wild-type strain were subjected to pull-down assay with GST/FcpA fusion proteins or GST and then probed with anti-FlaA2 antiserum by Western blotting (A). The same blot was stained with Ponceau S (B). (PDF) [file pone.0194923.s004.pdf]

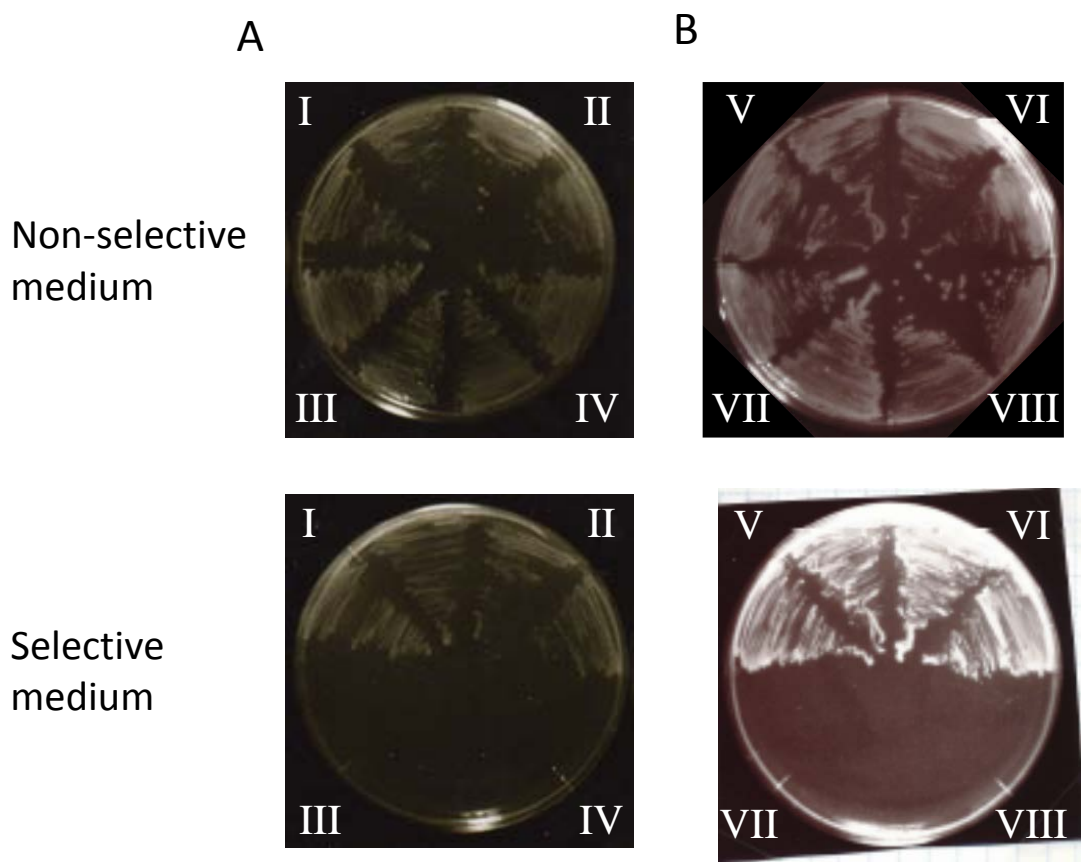

**S5 Fig. Two-hybrid analysis of the interaction between FcpA and FlaA2 (A) and FcpA and FlaB1 (B).**

Supplement: S5 Fig — Representative plates showing the growth of E. coli transformants. (I) bait (b): FlaA2; target (t): FcpA, (II) b: FcpA; t: FlaA2, (III) b: FlaA2; t: empty, (IV) b: FcpA; t: empty, (V) b: FlaB1; t: FcpA, (VI) b: FcpA; t: FlaB1, (VII) b: FlaB1; t: empty, (VI) b: FcpA; t: empty. (PDF) [file pone.0194923.s005.pdf]
